# Potentially toxic elements in the brains of people with multiple sclerosis

**DOI:** 10.1038/s41598-022-27169-9

**Published:** 2023-01-12

**Authors:** Roger Pamphlett, Michael E. Buckland, David P. Bishop

**Affiliations:** 1 Discipline of Pathology, Sydney Medical School, Brain and Mind Centre, The University of Sydney, Sydney, NSW Australia; 2 Department of Neuropathology, Royal Prince Alfred Hospital, Sydney, NSW Australia; 3 Hyphenated Mass Spectrometry Laboratory, School of Mathematical and Physical Sciences, University of Technology Sydney, Sydney, NSW Australia

**Keywords:** Multiple sclerosis, Environmental impact

## Abstract

Potentially toxic elements such as lead and aluminium have been proposed to play a role in the pathogenesis of multiple sclerosis (MS), since their neurotoxic mechanisms mimic many of the pathogenetic processes in MS. We therefore examined the distribution of several potentially toxic elements in the autopsied brains of people with and without MS, using two methods of elemental bio-imaging. Toxicants detected in the locus ceruleus were used as indicators of past exposures. Autometallography of paraffin sections from multiple brain regions of 21 MS patients and 109 controls detected inorganic mercury, silver, or bismuth in many locus ceruleus neurons of both groups, and in widespread blood vessels, oligodendrocytes, astrocytes, and neurons of four MS patients and one control. Laser ablation-inductively coupled plasma-mass spectrometry imaging of pons paraffin sections from all MS patients and 12 controls showed that combinations of iron, silver, lead, aluminium, mercury, nickel, and bismuth were present more often in the locus ceruleus of MS patients and were located predominantly in white matter tracts. Based on these results, we propose that metal toxicants in locus ceruleus neurons weaken the blood–brain barrier, enabling multiple interacting toxicants to pass through blood vessels and enter astrocytes and oligodendroglia, leading to demyelination.

## Introduction

Demyelination in multiple sclerosis (MS) is generally thought to be caused by a combination of genetic susceptibilities and environmental toxicants^1,2^. Advances have been made in finding genetic variants that predispose to MS, such as those involving the immune system^3–5^. However, environmental influences have been more difficult to pin down, with most attention being paid to low sun exposure or low vitamin D levels, cigarette smoking, and infection with Epstein-Barr virus^6–8^. However, no environmental agent has been found that can adequately account for the epidemiological, clinical, and pathological features of MS^9^.

**Figure 1:**
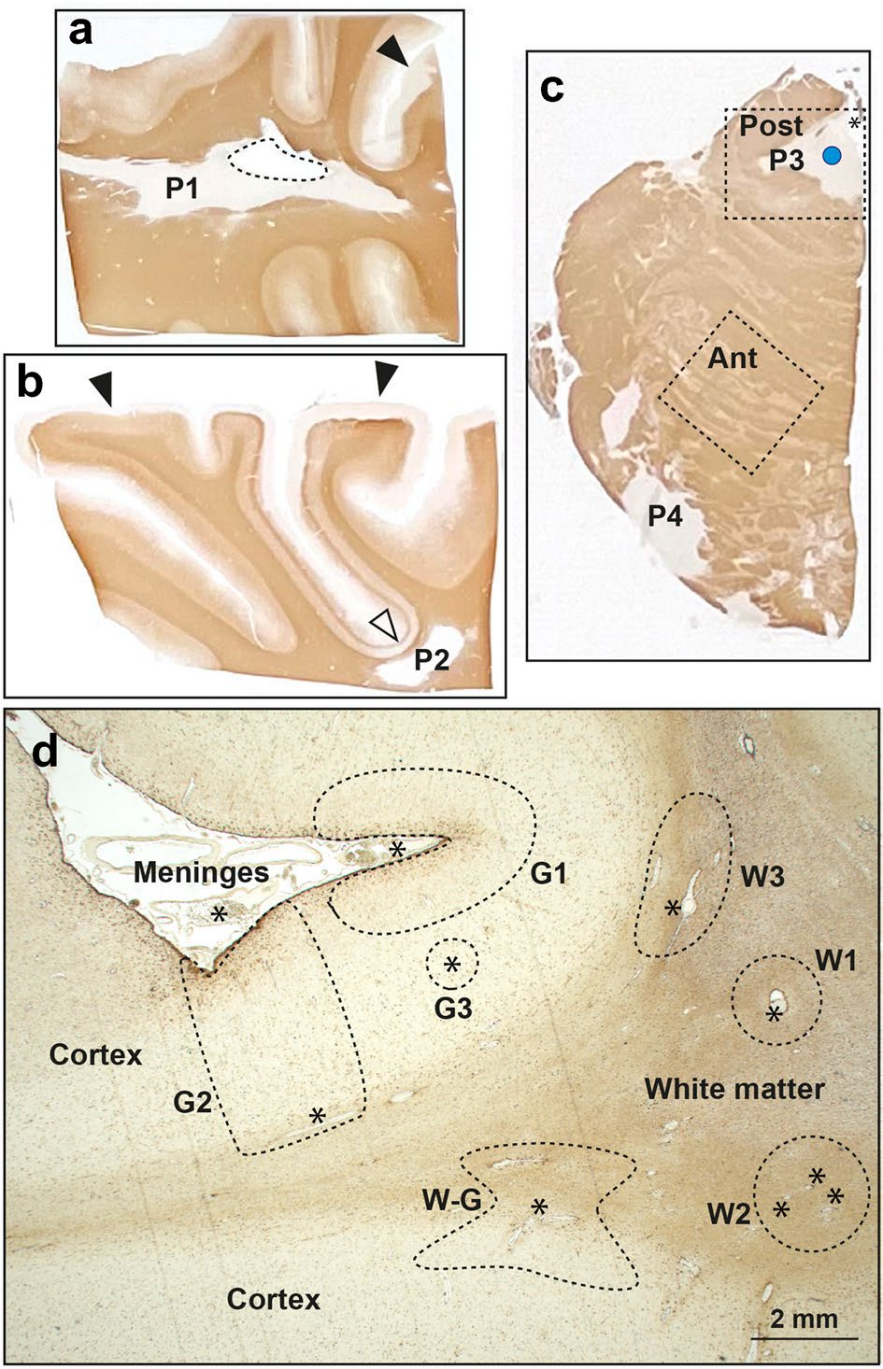
Sites of demyelination in MS (MBP stains myelin brown). (**a**) An MS plaque (P1) in the deep white matter surrounds the lateral ventricle (dashed outline). Subpial cortical demyelination is present in some gyri (arrowhead). MBP/H. MS09. (**b**) A subcortical white matter MS plaque (P2) is accompanied by cortical demyelination in the adjacent cortex at the base of the sulcus (open arrowhead). Widespread subpial cortical demyelination is present (closed arrowheads). MBP/H. MS09. (**c**) In this hemi-pons, one plaque is seen (P3) next to the fourth ventricle (*) and another with a broad base (P4) faces the meninges. The site of the locus ceruleus is shown by the blue dot. Typical sampling areas for LA-ICP-MSI are shown by the dashed boxes: posterior (Post) to include the locus ceruleus, and anterior (Ant) to include pale grey matter and more darkly staining white matter. MBP/H. MS09. (**d**) Asterisks indicate small blood vessels in the frontal lobe as centres for demyelination in either: W1 deep white matter from a single blood vessel; W2 deep white matter from multiple small blood vessels; W3 subcortical white matter from subcortical blood vessels; W-G leukocortical, from junctional white and grey matter blood vessels; G1 subpial, from leptomeningeal/subpial cortex blood vessels; G2 complete cortical, from combined leptomeningeal and grey/white junctional blood vessels; G3 intracortical from small blood vessels only seen microscopically. AMG/GFAP/H. MS14.

Many questions remain about the pathogenesis of MS. For an environmental contribution to MS to be considered credible, it needs to be able to explain recurrent episodes of demyelination, progression after the relapsing–remitting phase, the early age of onset, and an increasing incidence of the disease over time^9–11^. Pathological features that require explanation are the presence of demyelination in both white and grey matter (Fig. 1), the targeting of oligodendrocytes, damage to the blood–brain barrier, inflammation in MS plaques and in the meninges, astrocyte activation, the central role of blood vessels and their perivascular spaces, and the presence of oxidative stress, mitochondrial damage, and oligodendrocyte apoptosis^12–23^. Furthermore, it is not known why certain neurons are often damaged in MS, such as those in the locus ceruleus and thalamus^24–26^.

One group of toxic agents that could produce many features of MS are the potentially toxic elements (PTEs), formerly referred to as “heavy metals”, that include elements such as mercury, lead, and cadmium^27^. Many PTEs induce nervous system cytotoxicity, autoimmune inflammation, free radical formation, and blood–brain barrier damage^28–32^, all of which are implicated in MS. PTEs such as mercury and silver have been detected in the cells affected by MS, astrocytes, oligodendrocytes and neurons^33^, as well as in organelles such as mitochondria^34^. Mercury localises to the optic nerve^35,36^, often the first site affected by MS. Toxic metals are distributed widely in the human body^36–38^ and in some tumours^39^, and could therefore be responsible for extra-central nervous system manifestations associated with MS, such as hypertension, some cancers, retinal damage, and diabetes mellitus^11,24^. Increasing industrial output of PTEs, particularly from the burning of fossil fuels, means that growing amounts of metal toxicants are being released into the atmosphere, soil and water^40^, a rise that could underlie the increasing incidence of MS^9–11^. Several PTEs have previously been suspected to be involved with either the origin or progression of MS^41–49^.

Several difficulties arise when looking for the toxic initiators of pathological change in MS brains. Apart from iron, none of the PTEs can be detected in the living brain with current imaging techniques, so autopsied brains are needed to look for these elements. However, most people with MS live for many years after the initial onset of disease, and in these later years examination of autopsied MS brains is usually limited to gliotic plaques with little disease activity. In the few MS patients who die with recent plaques, the vigorous autoimmune inflammation obscures any initiating agents. The brain has efficient ways of removing toxicants from most cells^50^, so PTEs can be removed shortly after damaging these cells and will not be found at autopsy. MS-related PTEs are likely to be present in only small focal subsets of cells, so bulk chemical analyses are of little use in finding them. Finally, interactions between PTEs are known to augment their toxicity^51^, so multiple PTEs need to be detected simultaneously to assess the risk posed by synergistically acting toxicants.

To help overcome the difficulties, we looked for PTEs in the brains of people both with and without MS, using two elemental bio-imaging techniques. Autometallography is a histochemical technique that detects very low levels of inorganic mercury, silver, and bismuth within cells^52–55^. It has the advantage of being able to be used on large numbers of sections of brain which can be screened for widespread PTE uptake but has the disadvantage of being limited to these three inorganic metals. Laser ablation-inductively coupled plasma-mass spectrometry imaging (LA-ICP-MSI) on the other hand can simultaneously detect numerous elements in tissues^56^, though at a lower level of sensitivity than autometallography^57^.

We examined both MS plaques as well as non-involved grey and white matter sites within the brains to look for PTEs in regions not distorted by previous demyelination. In addition, we paid particular attention to the locus ceruleus in the pons, for three reasons. First, locus ceruleus neurons are unusually susceptible to PTE uptake and retain toxic metals for long periods of time, so elements found within locus ceruleus neurons can be used as a guide to past toxicant exposure^58^. Second, noradrenaline from the locus ceruleus is required to preserve the integrity of the blood–brain barrier^59^. The blood–brain barrier is focally disrupted early in MS^60^, and this disruption could be initiated by the uptake of toxic metals into individual locus ceruleus neurons^58,61^. Third, locus ceruleus neurons are damaged in MS^25^, which could be due to the accumulation of toxic metals in the locus ceruleus of people with MS^61^.

## Materials and methods

### Ethics

This study (X14-029) was approved by the Human Research Committee, Sydney Local Health District (Royal Prince Alfred Hospital Zone). All methods were carried out in accordance with relevant guidelines and regulations. The Human Research Committee, Sydney Local Health District (Royal Prince Alfred Hospital Zone) waived the need for written informed consent from relatives of individuals studied since this was a de-identified retrospective study of archived paraffin-embedded tissue. Data were fully anonymised on the research database after initial access to the records of the Multiple Sclerosis Research Australia Brain Bank and the New South Wales Department of Forensic Medicine.

### Sample collection

**Figure 2:**
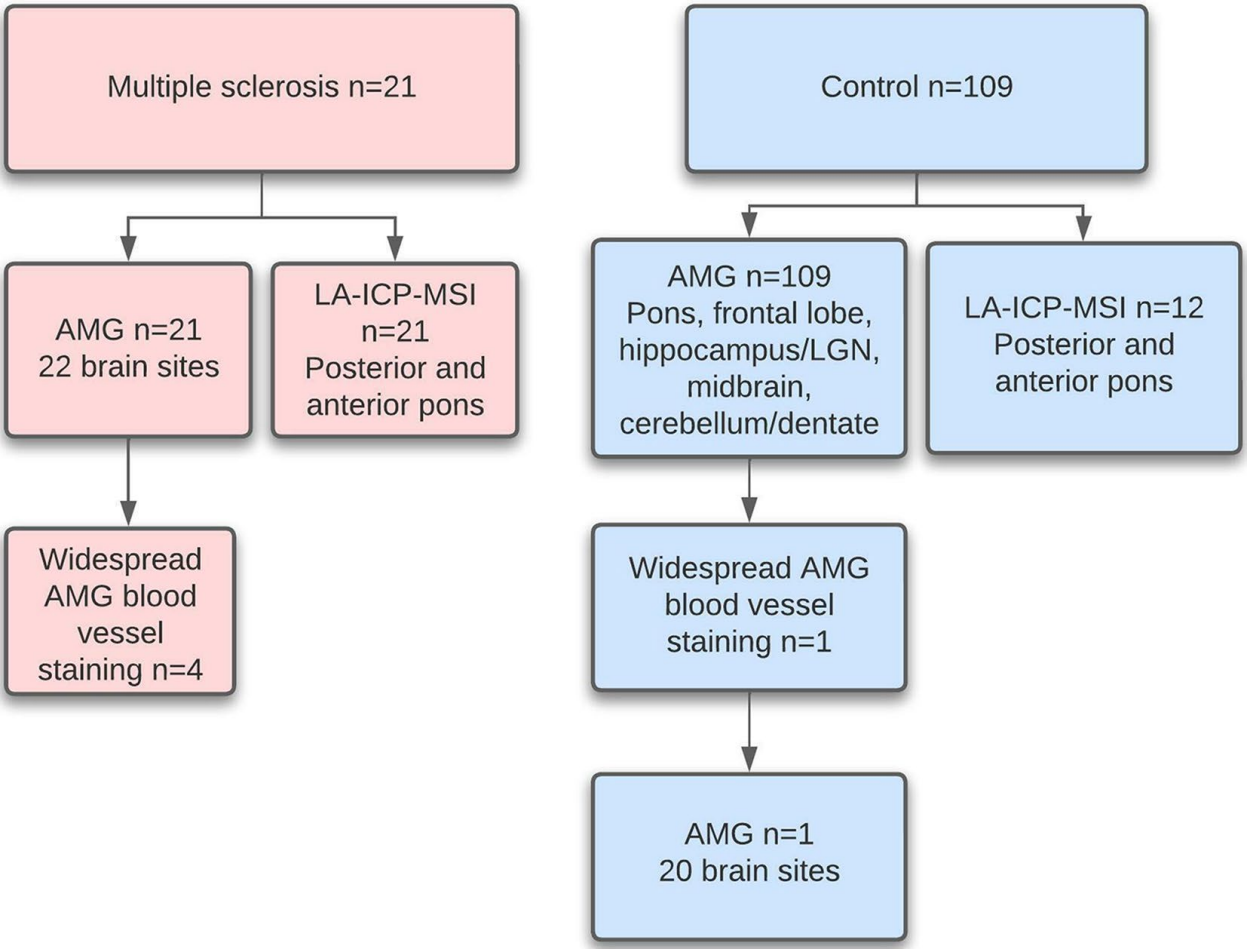
Sample collection and analysis. Twenty-one people with MS had autometallography (AMG) staining on paraffin sections from 22 different brain sites (including the pons). Four of these had widespread blood vessel AMG staining. All MS patients had laser ablation-inductively coupled plasma-mass spectrometry imaging (LA-ICP-MSI) of the posterior and anterior pons. The 109 non-MS controls had screening AMG of five brain regions. Only one of these had widespread AMG blood vessel staining, so this control had AMG performed on 20 different brain sites. Twelve controls had LA-ICP-MSI of the posterior and anterior pons.

Sample collection and analysis differed in MS patients and controls (Fig. 2).

#### Multiple sclerosis

Brain samples were available from 21 people (6 males, 15 females, mean age 61 years, SD 12 years, age range 36–84 years) who had been diagnosed by a neurologist as having MS and who had pre-donated their brains to the Multiple Sclerosis Research Australia Brain Bank (Table 1). The clinical diagnosis of MS was confirmed on macroscopic and microscopic examination of the brain by a neuropathologist (M. E. B). Twenty patients had multiple chronic demyelinated MS plaques, with an average time between MS diagnosis and death of 25 years (range 6–42 years, SD 11 years). One patient had acute and subacute plaques with a time between MS diagnosis and death of 0.4 years. Standard brain regions sampled were from the frontal, parietal, temporal and occipital lobes (cortex and underlying white matter), hippocampus (including the lateral geniculate nucleus), striatum, globus pallidus, thalamus, hypothalamus (including the mamillary body), cerebellar vermis and hemisphere (including the dentate nucleus), midbrain, rostral pons (including the locus ceruleus), and rostral medulla oblongata, as well as regions containing MS plaques; an average total of 22 sections per brain were taken (Fig. 2). Only hemi-blocks of the brain stem were available from most MS patients because fresh samples were sectioned mid-sagittally to allow for paraffin and frozen sections. Paraffin sections were stained with hematoxylin and eosin, Luxol-fast blue/cresyl violet (LFB/CV), myelin basic protein immunostaining/hematoxylin (MBP/H), and autometallography (AMG, see below). Selected blocks were stained with combined AMG and glial fibrillary acid protein immunostaining to detect metals within astrocytes.

#### Controls

Rostral pons paraffin blocks that contained the locus ceruleus were available from 109 people without MS (75 males, 34 females, mean age 54 years, SD 16 years, age range 28–104 years) who had autopsies performed at the New South Wales Department of Forensic Medicine. Known premortem medical conditions were none (N = 44), neurodegenerative disorder (N = 33), psychosis (N = 29), and one each of anorexia nervosa, epilepsy, and cancer. Paraffin sections from the rostral pons, frontal motor cortex, hippocampus (including the lateral geniculate nucleus), midbrain and cerebellar dentate nucleus were stained with AMG (Fig. 2). Only one control individual, a 37 year-old male with no known previous medical conditions who died suddenly, had widespread microvessels staining with AMG; in this individual, all paraffin sections from the same regions sampled in the MS patients were stained with AMG.

**Figure 3:**
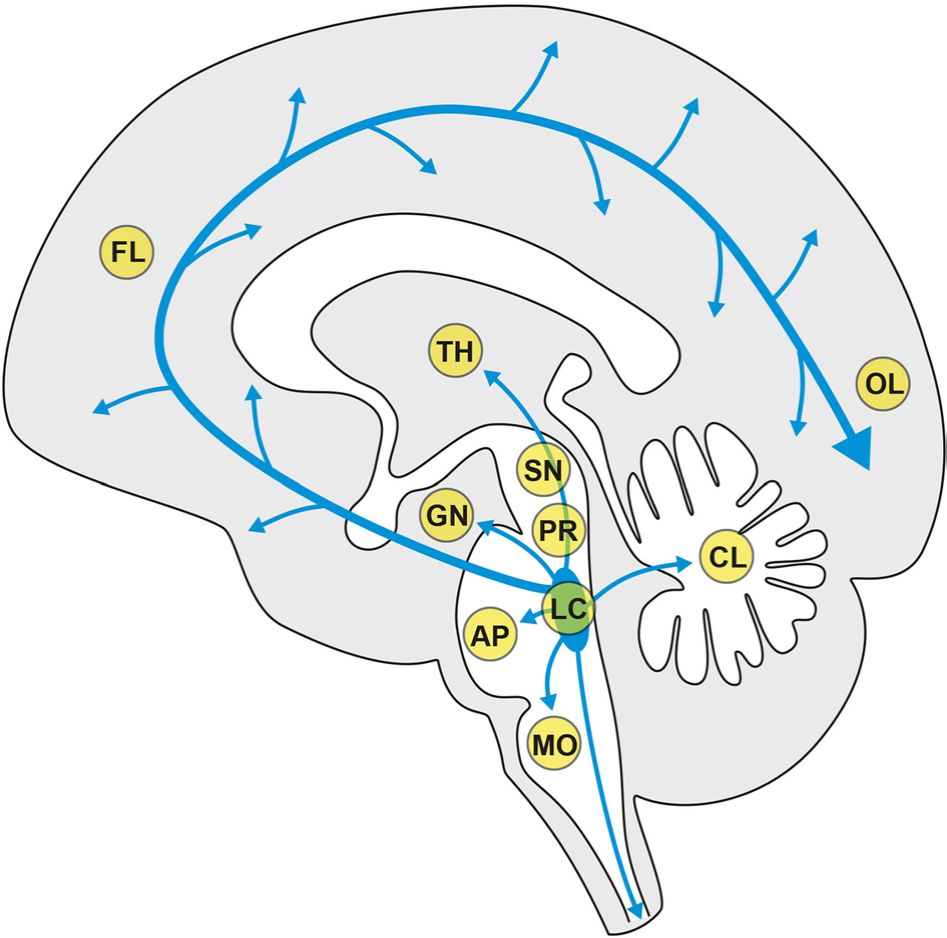
Location of brain regions where results are illustrated in the figures that follow. AP anterior pons (Figs. 4, 5, 6, 8, 9), CL cerebellum (Fig. 7), FL frontal lobe (Figs. 5, 6), GN lateral geniculate nucleus (Fig. 5), LC locus ceruleus (Figs. 4, 8, 9), MO medulla oblongata (Fig. 7), OL occipital lobe (Figs. 5, 6), PR pontine midline raphe (Fig. 7), SN substantia nigra (Fig. 4), TH thalamus (Figs. 5, 7). The blue arrows indicate the extent of locus ceruleus output of noradrenaline to the cerebrum, brain stem, cerebellum, and spinal cord. Diagram adapted from Bari et al. 2020^62^.

The locations of the anatomical regions of the brain that appear in the following figures are shown diagrammatically in Fig. 3, together with an indication of the widespread extent of locus ceruleus output of noradrenaline to microvessels, neurons, and glial cells within the nervous system^62,63^.

### Autometallography (AMG)

AMG is a sensitive amplification technique that detects as few as 10 susceptible metal-sulphide/selenide molecules in a cell^55^. Paraffin blocks were sectioned at 7 μm with a Feather S35 stainless steel disposable microtome blade, deparaffinised, and stained with silver nitrate AMG, which represents the presence of inorganic mercury, silver and bismuth as black silver grains surrounding these metals^52–54^. These three inorganic metals are referred to by the term “AMG-detected PTEs” (^AMG^PTEs). Sections were placed in physical developer containing 50% gum arabic, citrate buffer, hydroquinone, and silver nitrate at 26°C for 80 min in the dark, washed in 5% sodium thiosulphate to remove unbound silver, counterstained with mercury-free hematoxylin (H) or Luxol-fast blue (LFB), and viewed with bright-field microscopy. Sections were stained with hematoxylin only to act as a control for the AMG. Each staining run included a control paraffin section of mouse spinal cord where motor neuron cell bodies contained mercury following an intraperitoneal injection of mercuric chloride; these sections were from archived tissue blocks of a previously published experiment approved by the Animal Ethics Committee of the University of Sydney^64^.

Selected paraffin sections were stained with AMG and immunostained for astrocytes by incubation in polyclonal rabbit-anti-human glial fibrillary acidic protein (GFAP, DAKO Z0334) at 1:2000, visualised using 3,3 diaminobenzidine tetrahydrochloride and counterstained with hematoxylin. Astrocytes were identified by their GFAP-stained cell bodies and processes. Oligodendrocytes were identified by their GFAP-negativity, artefactually cleared cytoplasm, and contrast-enhanced nuclei^65^.

The percentage of locus ceruleus neurons containing 10 or more AMG grains was calculated using a 10 × 10 eyepiece grid viewed under × 400 magnification, as previously described^66^. These were graded as 0 (no neurons stained), 1 + (between 1 and 4% neurons stained), 2 + (between 5 and 20% neurons stained) or 3 + (> 20% neurons stained).

### Laser ablation-inductively coupled plasma-mass spectrometry imaging (LA-ICP-MSI)

To determine which ^AMG^PTEs AMG was demonstrating, since AMG can detect inorganic mercury, silver and bismuth, and to look for the presence of other PTEs, 7 μm paraffin sections of rostral pons from all 21 MS patients and from 12 controls (8 males, 4 females) were deparaffinised and subjected to LA-ICP-MSI (Table 1), with imaging of rectangular regions within the posterior and anterior pons (Fig. 1) for mercury (^201^Hg), silver (^107^Ag), bismuth (^209^Bi), aluminium (^27^Al), cadmium (^111^Cd), chromium (^52^Cr), cobalt (^59^Co), iron (^56^Fe), nickel (^60^Ni), and lead (^208^Pb), together referred to as ^MSI^PTEs. Phosphorus (^31^P) was imaged to estimate cellular density. Analyses were carried out on a New Wave Research NWR-193 laser hyphenated to an Agilent Technologies 7900 × ICP-MS, with argon used as the carrier gas. LA-ICP-MSI conditions were optimised on NIST 612 Trace Element in Glass CRM, and the sample was ablated with a 50 µm spot size and a scan speed of 100 µm/s at a frequency of 35 Hz. The data were collated into a single image file using in-house developed software^67^ and visualised using FIJI. The detection limits of LA-ICP-MSI are estimated to be between 0.05 and 0.81 μg per g^57^.

### Statistical analyses

GraphPad Prism 9 was used for statistical analyses. Fisher’s exact test was used for contingency testing. Significance was assessed at the two-tailed 0.05 level.

## Results

### Locus ceruleus autometallography

**Figure 4:**
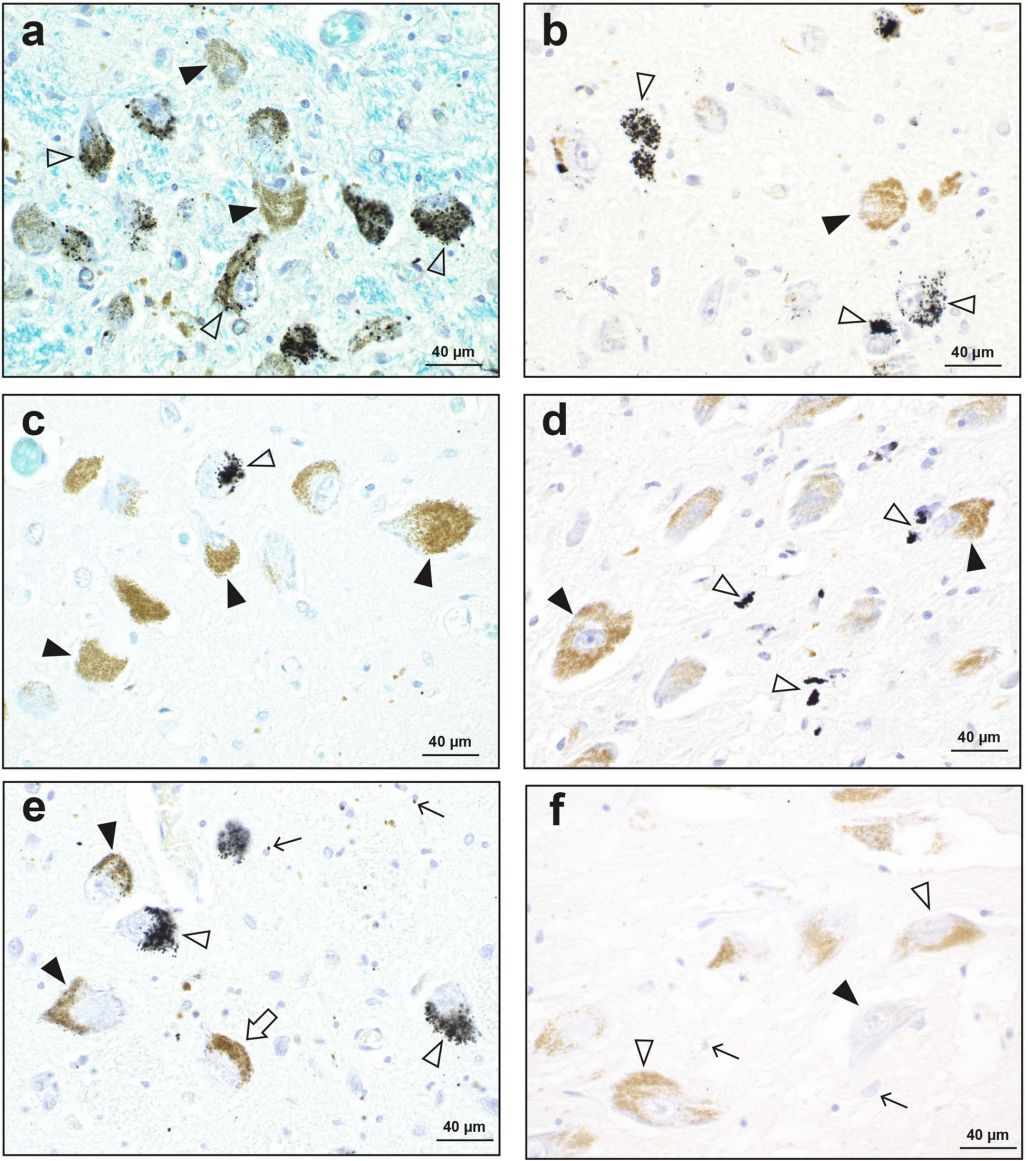
Autometallography of the locus ceruleus. (**a**) Black ^AMG^PTE granules are seen in the majority of locus ceruleus neurons (open arrowheads). Some locus ceruleus neurons contain no ^AMG^PTEs (closed arrowheads). MS18. AMG/LFB/CV. (**b**) Many locus ceruleus neurons contain ^AMG^PTE granules (open arrowheads). Some locus ceruleus neurons contain no ^AMG^PTEs (closed arrowhead). MS09. AMG/H. (**c**) A few locus ceruleus neurons contain ^AMG^PTEs (arrowhead). Most locus ceruleus neurons do not contain ^AMG^PTEs (closed arrowheads). MS14. AMG/LFB/CV. (**d**) Scattered black ^AMG^PTE granules (open arrowheads) are present in the neuropil of the locus ceruleus. Locus ceruleus neuronal cell bodies with brown neuromelanin granules (closed arrowheads) contain no ^AMG^PTEs. MS07. AMG/H. (**e**) In the locus ceruleus of a control, some neurons contain abundant ^AMG^PTEs, obscuring the neuromelanin (open arrowheads). Other neurons contain scattered ^AMG^PTEs within neuromelanin (closed arrowheads), and some are ^AMG^PTE-free (open arrow). Scattered oligodendrocytes (thin arrows) contain small ^AMG^PTE granules. CN12. AMG/H. (**f**) No ^AMG^PTEs are present in the substantia nigra compacta in the midbrain in the same brain as (**e**), either in pigmented neurons (open arrowheads), non-pigmented neurons (closed arrowhead), or glial cells (arrows). CN12. AMG/H.

^AMG^PTEs in locus ceruleus neurons ranged from a light sprinkling of fine AMG grains (mostly in neuromelanin-pigmented neurons) to heavy deposits that obscured the underlying neuronal structure (Fig. 4a–c,e). ^AMG^PTE content varied between individual locus ceruleus neurons. In one MS patient, ^AMG^PTEs were present in the neuropil between locus ceruleus neurons (Fig. 4d). ^AMG^PTEs were found in locus ceruleus neurons in similar proportions of MS patients (61%) and controls (57%). Similar proportions of MS patients and controls had different grades of ^AMG^PTEs in the locus ceruleus, with MS/control proportions being grade 0 39%/43%, grade 1+17%/16%, grade 2+13%/22%, and grade 3+22%/19%. A few substantia nigra compacta neurons in one MS patient, mostly non-pigmented, contained ^AMG^PTEs. No ^AMG^PTEs were seen in any control substantia nigra compacta neurons (Fig. 4f).

### Widespread brain autometallography

Widespread ^AMG^PTEs were seen in blood vessel walls, astrocytes, oligodendrocytes, and neurons in four out of 21 patients with chronic MS (17%) and on screening sections of one control out of 109 (0.9%), a significant difference (p = 0.003). This control therefore also had AMG performed on all available brain paraffin blocks.

#### MS patients

##### Blood vessels

**Figure 5:**
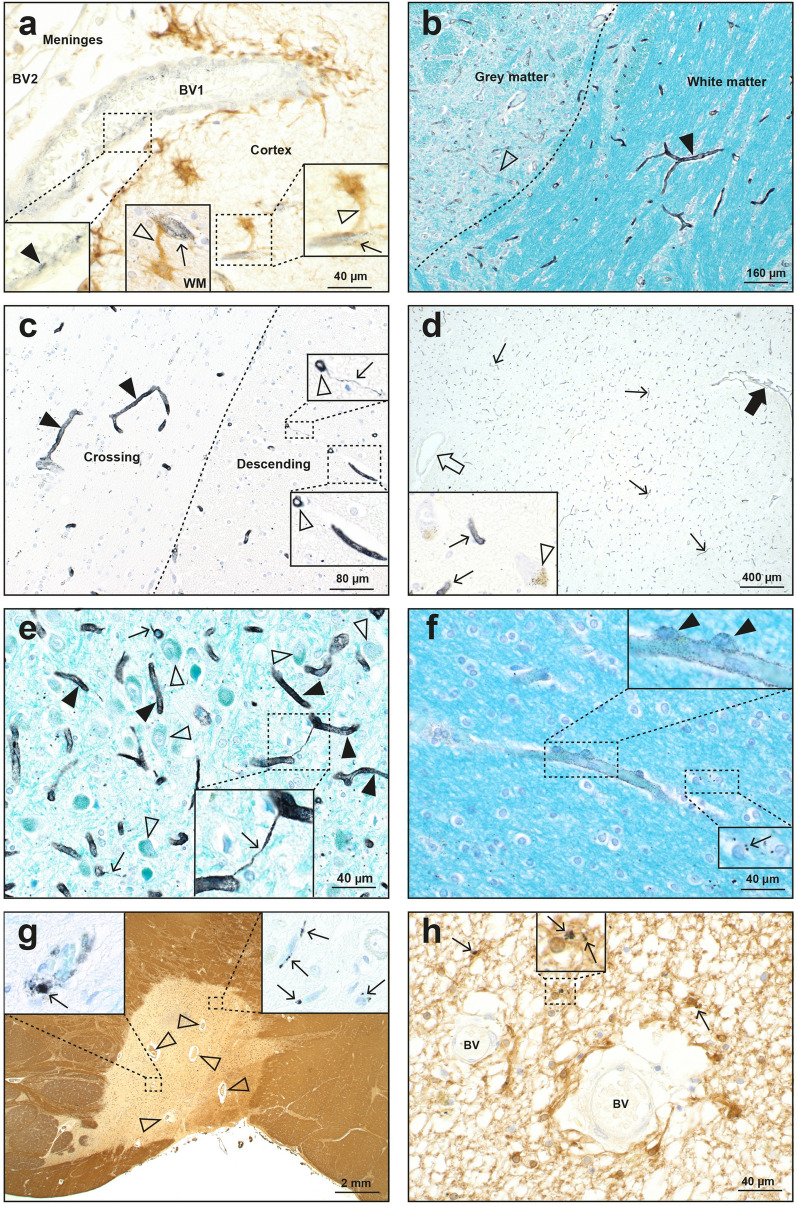
Autometallography of blood vessels. (**a**) A small leptomeningeal blood vessel (BV1), partially embedded in the superficial frontal cortex, contains ^AMG^PTEs in its wall (left inset, closed arrowhead). An adjacent larger leptomeningeal blood vessel (BV2) contains no ^AMG^PTEs. Right inset: a microvessel within the cortex contains ^AMG^PTEs (thin arrow), with an adjacent astrocyte cell body connected via a hypertrophic process (open arrowhead). Middle inset: in the white matter (WM) from the same patient, another ^AMG^PTE-containing microvessel (thin arrow) connects to an astrocyte cell body via a hypertrophic astrocytic process (open arrowhead). MS18. AMG/GFAP/H. (**b**) Microvessels (closed arrowhead) in the white matter of the anterior pons contain more ^AMG^PTEs than microvessels (open arrowhead) in the adjacent grey matter. MS18 AMG/LFB/CV. (**c**) Crossing white matter tracts in the anterior pons contain mostly horizontally arrayed microvessels with ^AMG^PTEs in their walls (closed arrowheads). In descending white matter tracts, the transversely sectioned microvessels indicate ^AMG^PTEs are present in the vessel walls (both insets, open arrowheads). In the upper inset an AMG-stained thread extends from one microvessel (arrow). MS18. AMG/H. (**d**) Numerous microvessels in the thalamus contain ^AMG^PTEs (thin arrows, also in enlarged inset). One larger blood vessel (open arrow), with a wall thickness of about 5 µm, does not contain ^AMG^PTEs. A smaller blood vessel, with a wall thickness of about 1 µm, contains ^AMG^PTEs (closed arrow). Inset: scattered thalamic neurons contain AMG grains within yellow–brown lipofuscin (open arrowhead). MS18. AMG/H. (**e**) Most microvessels in the lateral geniculate nucleus contain ^AMG^PTEs (closed arrowheads), but neuronal cell bodies (open arrowheads) do not. 1-µm-thick AMG-stained threads extend from several microvessels (thin arrows) and connect to adjacent microvessels (inset). MS18. AMG/LFB/CV. (**f**) In the frontal white matter, ^AMG^PTE deposits are seen in a microvessel wall and in surrounding pericytes (inset, closed arrowheads). Lower inset: scattered oligodendrocytes have small ^AMG^PTE deposits (arrow) adjacent to their nuclei. MS18. AMG/LFB/CV. (**g**) A chronic demyelinated MS plaque in the anterior pons contains numerous small blood vessels (open arrowheads) MBP/H. Left inset: scattered microvessels within the plaque have ^AMG^PTE-containing perivascular macrophages (arrow). Right inset: small ^AMG^PTE collections (arrows) are present in intra-plaque microvessels. Insets: AMG/H MS14. (**h**) A chronic demyelinated MS plaque in the occipital white matter has scattered astrocytes with small cytoplasmic ^AMG^PTE deposits (arrows, also in enlarged inset). Two small blood vessels (BV) within the plaque contain no ^AMG^PTEs. MS09. AMG/GFAP/H.

In the four MS patients with widespread brain ^AMG^PTEs, the ^AMG^PTEs were present in the walls of numerous brain microvessels, probably small venules and capillaries (Fig. 5). Regions with most ^AMG^PTE-containing microvessels were the cerebral leptomeninges, cerebral cortex and white matter, the thalamus, the lateral and medial geniculate nuclei, the cerebellar white matter, the anterior pons, and the inferior olivary nuclei in the medulla oblongata. Regions with only a few ^AMG^PTE-containing microvessels were the striatum and the cerebellar grey matter. In most regions (except for the thalamus and geniculate nuclei) ^AMG^PTE-containing microvessels were more prominent in white matter than in adjacent grey matter (Fig. 5b). Perivascular astrocytes often had hypertrophic connecting processes when the microvessel contained ^AMG^PTEs (Fig. 5a). ^AMG^PTEs could be seen surrounding pericytes adjacent to AMG-stained microvessels (Fig. 5f). Some small blood vessels in these four MS patients did not contain ^AMG^PTEs, even those near to vessels that did contain ^AMG^PTEs. The amount of microvessel ^AMG^PTEs varied between these four MS patients, but all had strong ^AMG^PTE staining in microvessels of the anterior pons and lateral geniculate nucleus.

**Figure 6:**
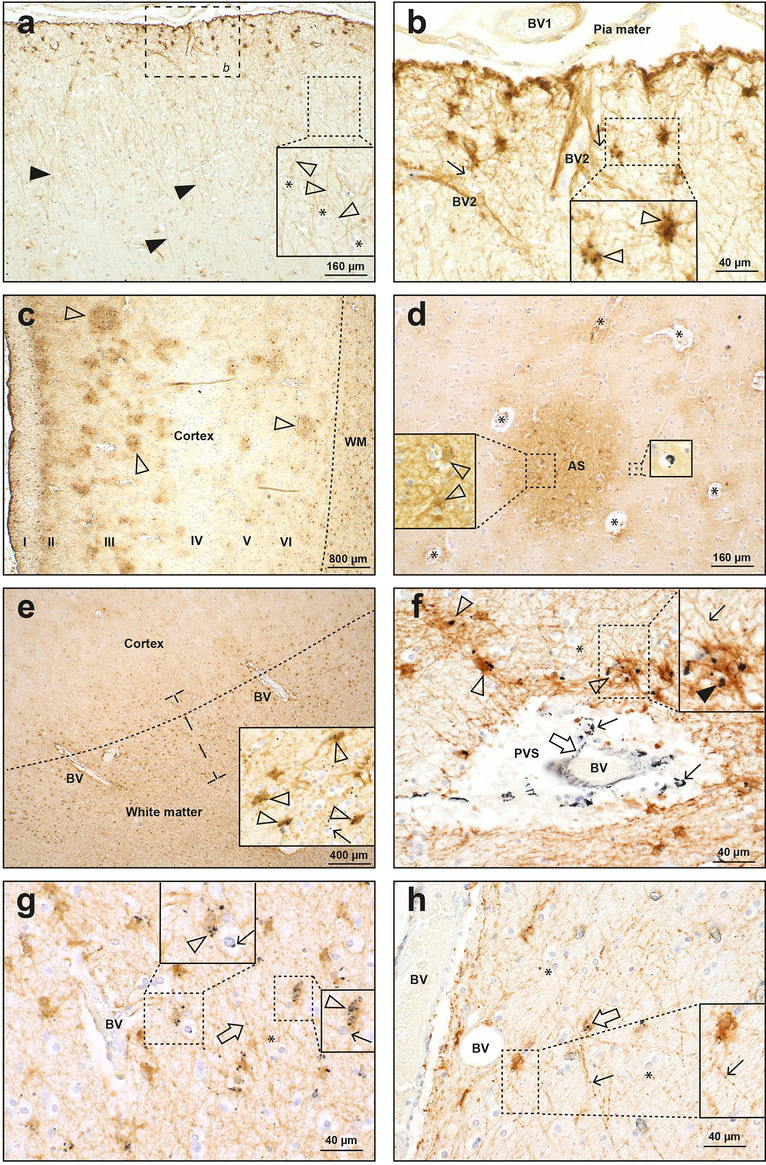
Autometallography of astrocytes and oligodendrocytes. (**a**) In the frontal lobe, cell bodies of subpial interlaminar astrocytes have dense brown GFAP staining (*b*: area shown at higher magnification in image **b**). The long undulating processes of interlaminar astrocytes (closed arrowheads) extend into the deeper layers of the cortex. Right inset: some interlaminar astrocyte processes (open arrowheads) appear to terminate on oligodendrocyte cell bodies (*). MS14. (**b**) A magnified view of the frontal lobe subpial cortex shows the cell bodies of interlaminar astrocytes containing black ^AMG^PTEs (inset, open arrowheads). Fine astrocytic processes (arrows) connect interlaminar astrocyte cells bodies with intracortical blood vessels (BV2). Meningeal (BV1) and intracortical blood vessels do not contain ^AMG^PTEs. MS14. (**c**) Multiple small foci of hypertrophic protoplasmic astrocytes (arrowheads), many with visible internal microvessels, are present in the frontal cortex, mostly in the outer cortical layers. Fibrous astrocytes in the underlying white matter (WM) have prominent GFAP staining. Roman numerals: cortical layers. MS11. (**d**) A group (AS) of hypertrophic protoplasmic astrocytes (left inset, open arrowheads) is present in the frontal cortex. Microvessels (*) surrounded by hypertrophic protoplasmic astrocytes are identifiable. Right inset: scattered cortical oligodendrocytes contain ^AMG^PTEs. MS09. (**e**) At the frontal lobe cortex-white matter junction (dashed line) reactive fibrous astrocytes are visible as brown dots, mostly in the subcortical white matter (within the bar). Right lower inset: many of these hypertrophic fibrous astrocytes contain ^AMG^PTE clusters (arrowheads). Scattered oligodendrocytes contain small paranuclear ^AMG^PTE deposits (thin arrow). Prominent blood vessels (BV) are present at the cortex-white matter junction. MS09. (**f**) In the white matter of the anterior pons, small ^AMG^PTE deposits (open arrow) are seen in the wall of a blood vessel (BV) and in surrounding macrophages (thin arrows) in the perivascular space (PVS). Fibrous astrocytes (open arrowheads) near the blood vessel contain ^AMG^PTEs. Inset: an astrocyte containing ^AMG^PTEs (closed arrowhead) sends long processes (thin arrow) away from the blood vessel, some of which appear to end on oligodendrocytes (*). MS09. (**g**) In the occipital white matter, glial cells adjacent to a blood vessel (BV) contain ^AMG^PTEs. Upper inset: multiple ^AMG^PTE granules (arrowhead) are present in a fibrous astrocyte abutting an oligodendrocyte with a small paranuclear ^AMG^PTE deposit (thin arrow). Right inset: an ^AMG^PTE-containing fibrous astrocyte (arrowhead) is closely adjacent to an oligodendrocyte (thin arrow). Several long astrocytic processes (open arrow) course through the white matter, some of which appear to end on oligodendrocytes (*). MS09. (**h**) In the white matter of the anterior pons, several astrocytes have the long beaded processes (thin arrows, and see inset) of polarised astrocytes, which pass close to, or appear to terminate on, oligodendrocytes (arrowheads). MS07. All AMG/GFAP/H.

Occasional small blood vessels in chronic MS plaques contained ^AMG^PTEs in endothelial cells or in macrophages within the blood vessel walls (Fig. 5g), and ^AMG^PTEs could also be seen in macrophages in perivascular spaces in unaffected white matter (Fig. 6f).

Some microvessels that contained ^AMG^PTEs had thin AMG-positive threads of uncertain origin which linked adjacent microvessels (Fig. 5c,e, Supplementary Fig. S1 online).

##### Astrocytes and oligodendrocytes

Clusters of hypertrophic protoplasmic astrocytes were often seen in the cerebral cortex, usually with central capillaries (Fig. 6c,d). Regions containing hypertrophic fibrous astrocytes were seen more diffusely in white matter (Fig. 6e). ^AMG^PTEs were found in interlaminar astrocyte cell bodies in subpial cerebral cortex layer I, often adjacent to larger venules in the leptomeninges (Fig. 6a,b). ^AMG^PTEs were also present in large numbers of fibrous astrocytes in subcortical cerebral white matter (Fig. 6e), olivary white matter in the medulla oblongata, and in cerebellar dentate white matter. Long polarised astrocytes in white matter surrounding blood vessels, some of which appeared to terminate on oligodendrocytes, often contained ^AMG^PTEs (Fig. 6h). Fibrous astrocytes containing ^AMG^PTEs could be seen adjacent to either ^AMG^PTE-positive or negative blood vessels (Fig. 6f,g). A few scattered fibrous astrocytes in chronic MS plaques contained ^AMG^PTEs (Fig. 5h).

Oligodendrocytes near to microvessels containing ^AMG^PTEs in white matter throughout the brain often contained perinuclear ^AMG^PTE deposits (Figs. 4e, 5f, 6d,g). Fewer oligodendrocytes in the cerebral cortex contained ^AMG^PTEs deposits (Fig. 6d).

##### Neurons

**Figure 7:**
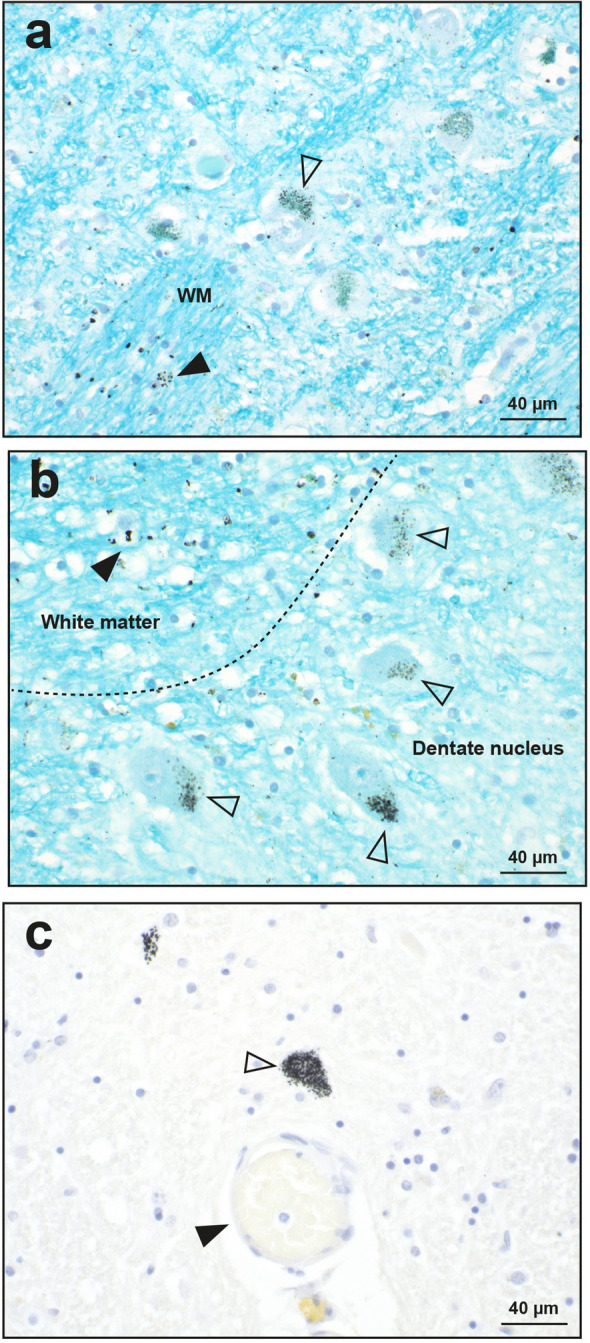
Autometallography of neurons. (**a**) Scattered thalamic neuronal cell bodies (open arrowhead) contain ^AMG^PTE grains. White matter (WM) tracts within the thalamus have numerous ^AMG^PTE-containing glial cells (closed arrowhead). AMG/LFB/CV. MS09. (**b**) Many neurons in the dentate nucleus of the cerebellum contain ^AMG^PTE granules (open arrowhead). The adjacent cerebellar white matter has numerous glia containing ^AMG^PTEs (closed arrowhead). AMG/LFB/CV. MS09. (**c**) Pontine midline raphe neurons contain abundant ^AMG^PTEs (open arrowhead). A nearby blood vessel (closed arrowhead) is ^AMG^PTE-free. MS09. AMG/H.

In the four MS patients where widespread microvessels contained ^AMG^PTEs, neurons containing ^AMG^PTEs (apart from the locus ceruleus) were seen in the thalamus (Fig. 7a), the cerebellar dentate nucleus (Fig. 7b), and the pontine midline raphe nucleus (Fig. 7c).

#### Control

In only one control brain were widespread blood vessel, glial and neuronal ^AMG^PTEs detected, in a similar distribution to that of the four MS patients above (Table 1, Supplementary Fig. S1 online). ^AMG^PTEs were prominent in microvessels in the anterior pons, lateral geniculate nucleus, and cerebral and cerebellar white matter, in white matter astrocytes and oligodendrocytes, and in neurons in the thalamus and dentate nucleus, but not in substantia nigra neurons (Fig. 4f). LA-ICP-MSI of the pons in this control brain (see below) indicated that the PTE present was silver only.

#### LA-ICP-MSI

Details of the individuals who had LA-ICP-MSI of the pons are listed in Table 1. All LA-ICP-MSI results are shown in Supplementary Fig. S2 online for MS patients and Supplementary Fig. S3 online for controls, with results summarised in Table 1 and Fig. 10.

#### MS patients

##### Posterior pons

**Figure 8:**
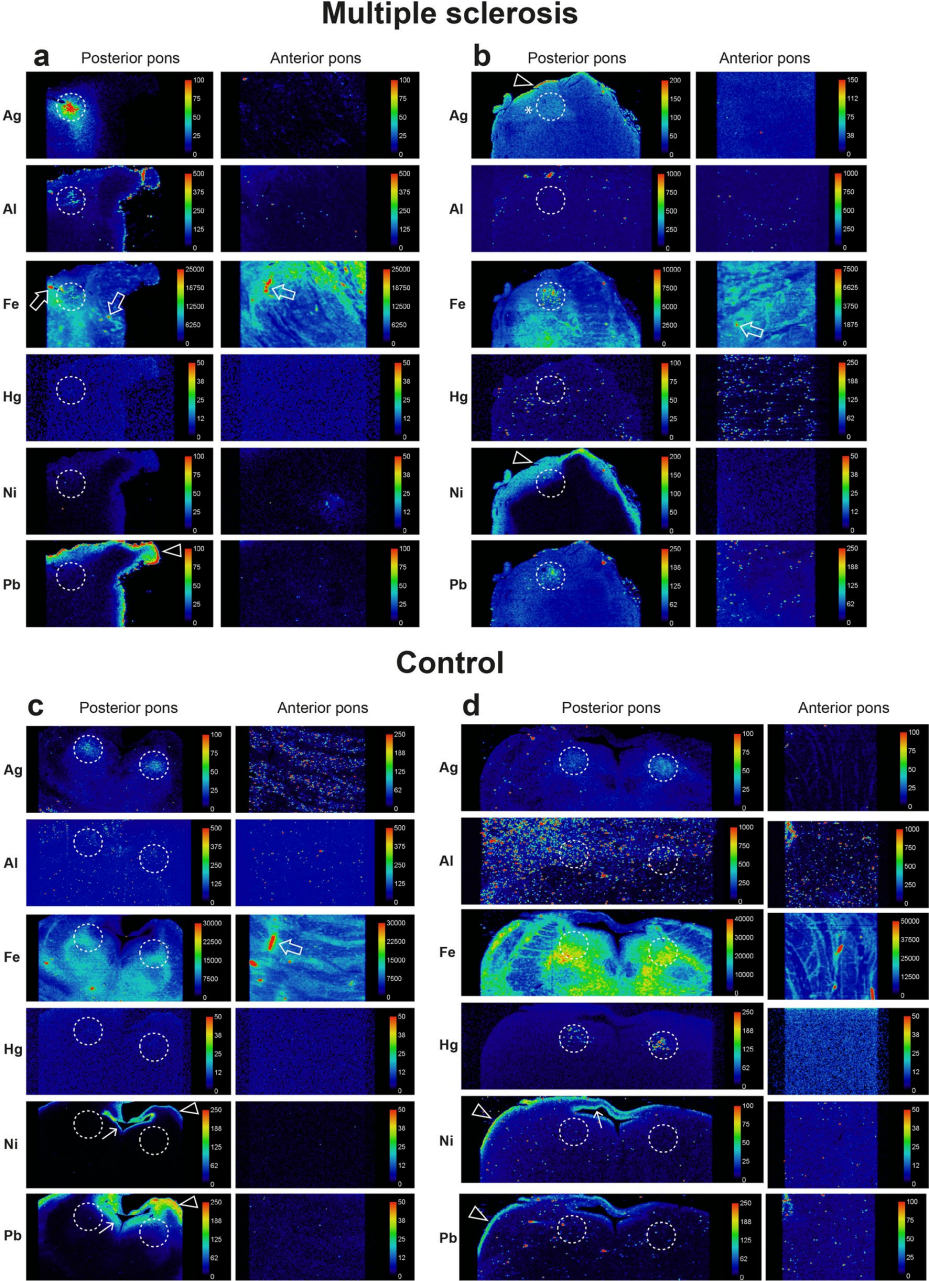
Representative images of LA-ICP-MSI in MS pons and control samples I. The position of the locus ceruleus is within the dashed circles. (**a**) MS05. *Posterior pons*: locus ceruleus ^MSI^PTEs are silver, aluminium, and iron. Iron is also present in non-locus ceruleus regions (green), and around small blood vessels (arrows). Lead is seen in the leptomeninges and subpial regions (arrowhead). *Anterior pons*: iron in present in linear white matter tracts, and both in (red) and around (green) blood vessels (arrow). Linear silver is present. (**b**) MS14. *Posterior pons*: locus ceruleus ^MSI^PTEs are iron, nickel (focal), and lead. Iron (green) is also present in non-locus ceruleus regions. Silver and nickel are seen in subpial regions (arrowheads), with possible penetration of silver into deeper tissue (*). *Anterior pons*: iron and mercury are present in linear regions, with iron in and around blood vessels (arrow). (**c**) CN12. *Posterior pons*: locus ceruleus ^MSI^PTEs are silver and iron. Iron (green) is also present in extra-locus ceruleus regions, and around blood vessels (arrow). Lead and nickel are seen in subpial (arrowheads) and subependymal (thin arrow) regions. *Anterior pons*: Iron is present in linear regions and in and around blood vessels (arrow). Linear iron and silver are present. (**d**) CN05. *Posterior pons*: locus ceruleus ^MSI^PTEs detected are silver, iron, and mercury. Iron (green) is also present in extra-locus ceruleus regions. Lead and nickel are seen in subpial regions (arrowheads) and nickel in subependymal regions (thin arrow). *Anterior pons*: Iron in present in linear white matter tracts and in and around blood vessels (arrow). Linear deposits of silver are seen.

**Figure 9:**
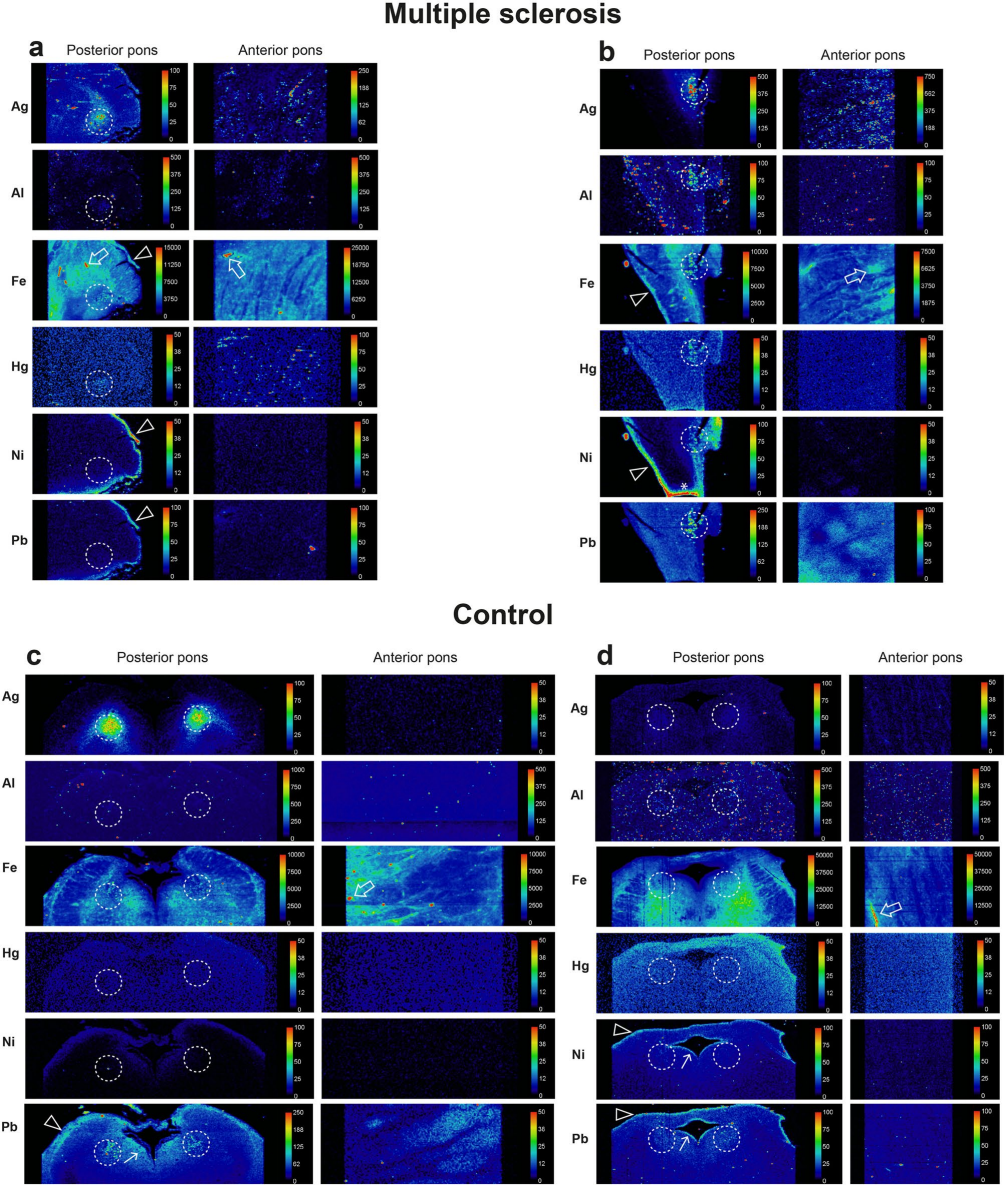
Representative images of LA-ICP-MSI in MS and control pons samples II. The position of the locus ceruleus is within the dashed circles. (**a**) MS09. *Posterior pons*: locus ceruleus ^MSI^PTEs are silver, iron, and mercury. Iron is also present in non-locus ceruleus regions (green), and in and around small blood vessels (arrow). Iron, lead, and nickel are seen in the leptomeninges and subpial regions (arrowheads). *Anterior pons*: iron and mercury are present in linear regions (possibly blood vessels), and iron in and around blood vessels (arrow). **(b)** MS18. *Posterior pons*: locus ceruleus ^MSI^PTEs are silver, aluminium, iron, mercury, nickel, and lead. Iron is also present in non-locus ceruleus regions (green). Iron and nickel are seen in subpial regions (arrowheads), with nickel on a cut surface (*). *Anterior pons*: silver, iron and lead are present in linear regions, and iron (green) around blood vessels (arrow). (**c**) CN08. *Posterior pons*: locus ceruleus ^MSI^PTEs are silver, iron, and lead. Iron is present in extra-locus ceruleus regions. Lead is seen in subpial (arrowhead) and subependymal (thin arrow) regions. *Anterior pons*: Iron in present in linear regions and around blood vessels (arrow). Linear deposits of iron and lead are present. (**d**) CN02. *Posterior pons*: locus ceruleus ^MSI^PTEs are iron and lead. Iron is also present in extra-locus ceruleus regions (green). Small amounts of lead and nickel are seen in subpial and subependymal regions (thin arrows). *Anterior pons*: Linear silver and iron, and iron in and around blood vessels (arrow), are present.

^MSI^PTEs detected in the locus ceruleus of the 21 MS patients were iron (n = 21, 100%), silver (n = 16, 76%), lead (n = 15, 71%) aluminium (n = 6, 29%), mercury (n = 5, 24%), nickel (n = 5, 24%), and bismuth (n = 4, 19%) (Table 1, Figs. 8 and 9, Supplementary Fig. S2 online). No cobalt, cadmium or chromium was detected. Iron was also seen within the lumens of blood vessels (due to iron in red blood cells) and surrounding blood vessels. All MS patients had at least one non-iron metal in the locus ceruleus, and combinations of PTEs were common (Table 1). Overall, 86% of MS patients had two or more non-iron ^MSI^PTEs in the locus ceruleus, with individual proportions being 52% (two ^MSI^PTEs), 14% (three ^MSI^PTEs), 14% (four ^MSI^PTEs) and 5% (five ^MSI^PTEs). The most common combination, in 43% of patients, was silver and lead. Lead and nickel were seen in some samples in either the leptomeninges or adjacent subpial region, or in the subependymal region surrounding the 4th ventricle.

##### Anterior pons

Iron was detected in irregular linear arrays in the anterior pons of all 21 patients, most commonly in white matter tracts (Table 1, Figs. 8 and 9, Supplementary Fig. S2 online) and surrounding some blood vessels. 75% of MS patients had at least one one-iron metal in the anterior pons. Silver was the most detected non-iron metal in the anterior pons (57% of patients), followed by lead (43%), and aluminium and mercury (10% each). Some thin linear arrays of ^MSI^PTEs in the anterior pons (eg, silver and mercury in MS09, Fig. 9a) may correspond to the ^AMG^PTEs seen in blood vessels in these samples.

#### Controls

##### Posterior pons

^MSI^PTEs detected in the locus ceruleus of the 12 controls were iron (100%), silver (58%), lead (33%), mercury (25%), and aluminium (17%) (Table 1, Figs. 8 and 9, Supplementary Fig. S3 online). No bismuth, nickel, cobalt, cadmium, or chromium was detected. Iron was also seen anterior to the locus ceruleus and surrounding some blood vessels. Overall, 42% of controls had two or more non-iron ^MSI^PTEs in the locus ceruleus (Table 1). The most common combinations were silver and lead (25%) and silver and mercury (25%). Proportions of controls with two or more non-iron ^MSI^PTEs in the locus ceruleus were 25% (two ^MSI^PTEs), and 17% (three ^MSI^PTEs). When both locus ceruleus nuclei were present, asymmetry of PTE content was sometimes seen between nuclei (Supplementary Fig. S3 online). Some ^MSI^PTEs, notably lead and nickel, were seen in the leptomeninges or regions adjacent to cerebrospinal fluid, in subpial and subependymal tissue.

**Table 1:**
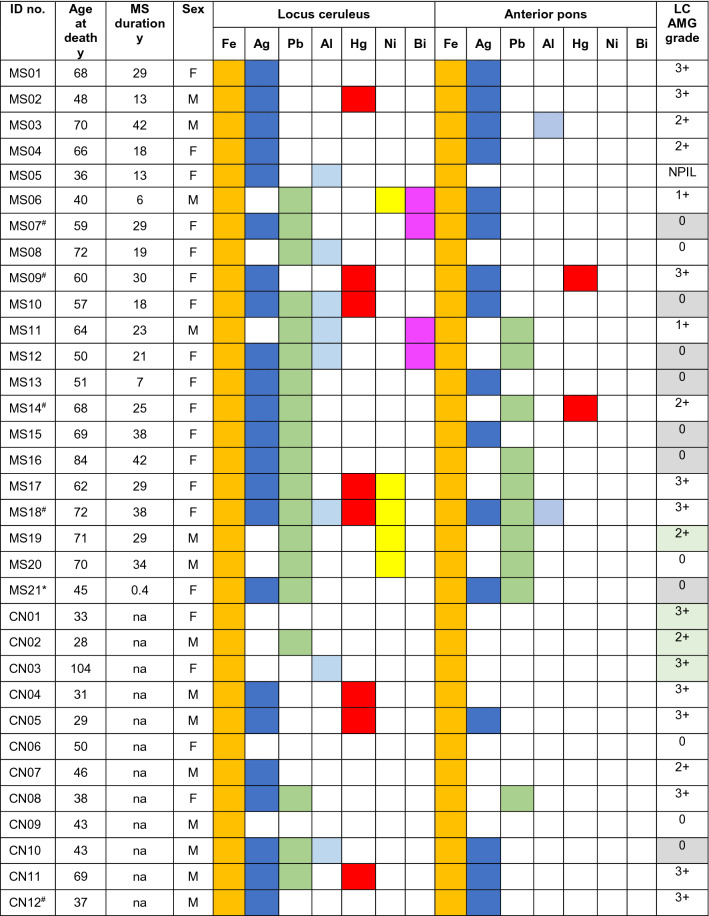
Potentially toxic elements detected on LA-ICP-MSI in the locus ceruleus and anterior pons.

In the column on the right, grey shading indicates samples that are AMG negative but LA-ICP-MSI positive for Ag, Hg, or Bi; green shading indicates samples that are AMG positive but LA-ICP-MSI negative for Ag, Hg and Bi.

*LC* locus ceruleus, *AMG* autometallography, *CN* control, *MS* multiple sclerosis, *NPIL* neuropil, *na* not applicable.

*Subacute MS (others chronic).

^#^Widespread AMG-detected metals.

##### Anterior pons

Iron was detected in irregular linear arrays in the anterior pons of all 12 controls, mostly in white matter (Table 1, Figs. 8 and 9, Supplementary Fig. S3 online), and surrounding some blood vessels. 42% of controls had one non-iron metal in the anterior pons. No combinations of two or more non-iron ^MSI^PTEs were seen. Silver was the most detected non-iron metal in the anterior pons (33%), followed by lead (8%). Some thin linear arrays of PTEs in the anterior pons (e.g., silver in CN12, Fig. 8c) may correspond to the ^AMG^PTEs seen in pontine blood vessels in these samples.

#### Comparisons of LA-ICP-MSI findings between MS patients and controls

##### PTEs in the locus ceruleus

**Figure 10:**
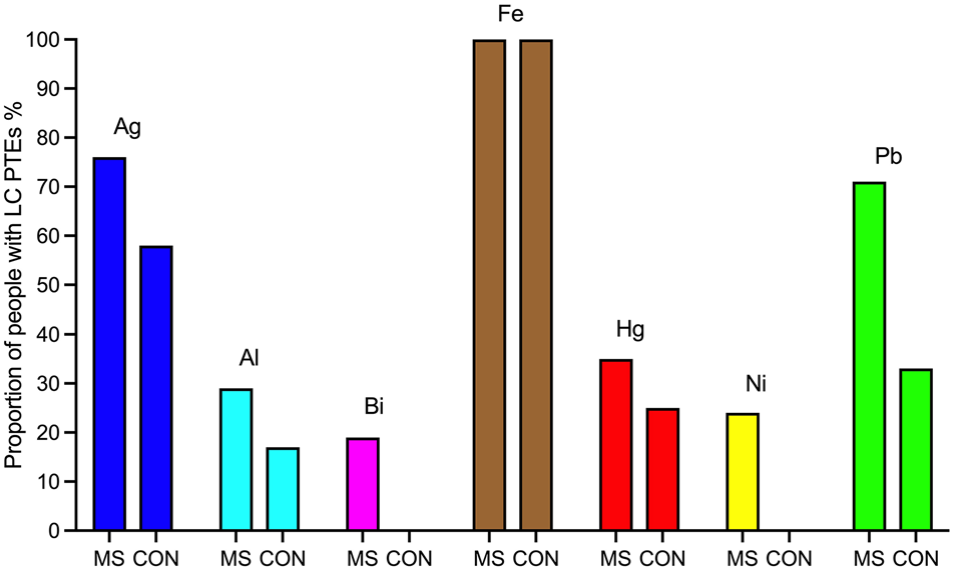
The proportion of people with potentially toxic elements (PTEs) detected in the locus ceruleus with LA-ICP-MSI. All individuals had iron in the locus ceruleus. Similar proportions of MS patients and controls (CON) had silver and mercury in the locus ceruleus, while slightly more MS patients had locus ceruleus aluminium and lead. Bismuth and nickel were present in the locus ceruleus of a few MS patients, but not in controls.

The locus ceruleus of MS patients and controls contained similar proportions of silver, mercury, and aluminium (Table 1, Fig. 10). MS patients had a slightly greater proportion of locus ceruleus lead than controls. Bismuth and nickel were seen only in the locus ceruleus of MS patients (Fig. 10). A greater proportion of MS patients (78%) had more than one non-iron PTEs in the locus ceruleus than controls (42%) (p = 0.016) (Table 1).

##### PTEs in the anterior pons

A greater proportion of MS patients (90%) had one or more non-iron PTE in the anterior pons white matter than controls (42%) (p < 0.0001) (Table 1).

##### Discordant AMG/LA-ICP-MSI findings

In eight people, locus ceruleus AMG was negative but LA-ICP-MSI detected ^AMG^PTEs (silver, mercury and/or bismuth) (Table 1) suggesting these ^MSI^PTEs were not in their inorganic forms in these samples. In four people, AMG detected locus ceruleus inorganic mercury, silver, or bismuth where LA-ICP-MSI did not find these metals (Table 1). This is likely to be because low levels of these metals could be detected by AMG, but not by the less sensitive LA-ICP-MSI^55,57^.

## Discussion

Key findings of this study are that people with MS are more likely than non-MS controls to have widespread deposits of potentially toxic elements in their brains, and that combinations of toxic metals are present more often in MS brains than in controls. Not all people with toxic metals in their brains had MS, suggesting that susceptibilities to toxic metal-induced autoimmune inflammation are required to precipitate demyelination.

Seven PTEs were detected in the locus ceruleus of MS and control brains, indicating previous exposure to these elements. Some of these PTEs were also seen in the white matter of the anterior pons, more often in people with MS. These PTEs share the toxic properties of increasing oxidative stress, promoting autoimmunity and inflammation, damaging mitochondria, impairing the blood–brain barrier, and enabling apoptosis^30,31^, all features thought to play parts in the pathogenesis of MS^9^.

*Iron* has been implicated in the pathogenesis of both the relapsing–remitting and progressive forms of MS and is found at high levels in normal oligodendrocytes^68–70^. Iron was seen in all locus ceruleus neurons in our samples, as previously reported^61^. The ability of iron to generate free radicals could be a reason why the locus ceruleus is susceptible to neuronal loss in neurological disorders such as Parkinson’s and Alzheimer’s diseases^71^, and locus ceruleus damage has been found in MS^25^. Iron was present in some white matter tracts in our samples, and can be demonstrated in white matter on brain magnetic resonance imaging^72^, probably because of the high iron content in oligodendrocytes^73,74^. The perivascular iron seen in our samples has been noted before^75^, and could be due to damage to small blood vessels from contained PTEs.

*Silver* was the most common non-iron PTE detected in the locus ceruleus of both MS patients and controls. Silver and lead combinations were found more often in the locus ceruleus of MS patients than controls. The high frequency of silver-containing cells in the locus ceruleus is the main reason locus ceruleus autometallography was so often positive in both MS patients and controls. Silver was localised to some white matter tracts in the anterior pons, especially in MS patients, and was the only non-iron PTE detected in the control who had widespread brain PTE deposits. Little attention has been paid to the possibility that silver could play a part in MS, despite the fact that silver is a neurotoxin^76,77^ that can disrupt the blood–brain barrier^78–80^ and damage myelin^81,82^. Silver and mercury often co-exist in the brain^55,83^, and silver toxicity is increased in the presence of nickel^84^, so finding silver-nickel combinations in MS brains suggests they could play a role in demyelination. It has been proposed that sensitivity to silver-induced autoimmunity is involved in several diseases, such as fibromyalgia and connective tissue disorders^85,86^, so investigating MS patients for sensitivity to toxic metals such as silver may be of value. Common sources of silver exposure are dental amalgams, and silver nanoparticles that are used in healthcare, food, textiles, and electrical products^87^. Of note, silver nanoparticles can be detected with LA-ICP-MSI^88^.

Increased blood *lead* levels have been proposed to augment the risk of MS^89^. After silver, lead was the most common non-iron PTE detected in our locus ceruleus samples and was often present in the anterior pons white matter. Lead neurotoxicity damages systems involved in MS, since it affects DNA binding proteins that control myelin basic protein, decreases the activity of CNPase required for myelin synthesis, disrupts the blood–brain barrier, induces oxidative stress, binds to enzymes with sulfhydryl groups and renders them non-functional, accumulates in and damages mitochondria, has toxic effects on oligodendrocytes and astrocytes, substitutes for calcium and zinc, and initiates apoptosis^90^. Common sources of lead exposure are leaded petrol and lead-based paint (in high-income countries), and industrial processes such as battery manufacture, smelting, mining, and coal combustion (in lower-income countries)^91,92^.

*Aluminium* was present in the locus ceruleus in a similar proportion of MS patients and controls. Aluminium levels in brain tissue have been reported to be high in MS^93–95^. Aluminium is a neurotoxin that increases autoimmunity, and human exposure is common due to its presence in drinking water, food additives, cosmetics, and pharmaceutical products such as vaccine adjuvants^96^.

*Mercury* was detected in the locus ceruleus in a similar proportion in MS patients and controls, but in white matter of more MS patients than controls. Most proposals that mercury could play a role in MS have been based on reports implicating mercury-containing dental amalgam restorations in MS^41^. The US Food and Drug Administration has recommended that people with pre-existing neurological disease, including MS, are provided with non-mercury dental restorations^97^. This recommendation noted that the weight of existing evidence does not definitely show that exposure to mercury from dental amalgam leads to adverse health effects, but mentions the contradictory findings from different studies, exposure to additional amounts of mercury from fish consumption, the role of the body’s ability to convert one form of mercury into another^98^, and the challenges in defining a threshold of toxicity for chronic low-level mercury exposure. In a man who injected himself with metallic mercury, mercury was detected in astrocytes, oligodendrocytes, and locus ceruleus neurons^33^, all cells affected in MS^9,25^. Toxic metals such as mercury can initially be detected in locus ceruleus neurons in people aged in their twenties^58^, an age at which the first symptoms of MS commonly appear^9^. Varying levels of mercury have been detected in on X-ray fluorescence microscopy in individual locus ceruleus neurons in the brains of people with MS, suggesting these neurons may play a role in determining the multiple sites of demyelination^61^. Organic mercury, which readily crosses the blood–brain barrier, is slowly demethylated to more toxic inorganic mercury which persists in the brain^98–100^, a process that could contribute to progressive MS, since the inorganic form can be the more toxic form of the metal^100^. Toxic actions of mercury that parallel those thought to operate in MS are autoimmune inflammation^101^, apoptosis and free radical formation^102^, mitochondrial dysfunction^103^, and damage to the blood–brain barrier^104^.

*Nickel* was seen in the locus ceruleus in a few MS patients, but not in controls. Serum levels of nickel have been reported to be higher in MS patients than in controls^105^. Mitochondrial dysfunction and oxidative stress contribute to the neurotoxicity of nickel^106,107^. As mentioned, nickel can interact with metals such as silver to increase overall neurotoxicity^84^. Nickel is used in many industrial metallurgical processes and in nanoparticles in various fields^107^.

*Bismuth*, seen in the locus ceruleus of a few patients with MS but not in controls, has been detected previously in neurons, glia, and blood vessels within human brains^108^, and can affect gene expression in macrophages^109^. The cellular distribution of bismuth closely parallels that of mercury^110^, which could facilitate toxic interactions between these two metals.

Synergistic interactions between PTEs, and interactions of PTEs with essential elements, are increasingly being recognised^51,111^. Our MS patients were more likely than controls to have combinations of PTEs in their brains. Of note, the control who had widespread ^AMG^PTE uptake in his brain had only silver detected in his locus ceruleus on LA-ICP-MS, raising the possibility that he did not suffer from MS because of an absence of toxicant interactions. Other possibilities, however, are that he had no predisposing factors to toxicant-induced MS, or that because of his early age of death he avoided getting MS that he could have developed had he lived longer.

Autometallography-detected metals in this study were prominent in brain microvessels, mostly those within white matter, as well as in astrocytes surrounding these microvessels. A primary role for blood vessels in the pathogenesis of MS has long been recognised, since veins are present at the centre of white matter plaques, and these veins continue within demyelinated "Dawson’s fingers" that extend from the ventricles^112,113^. Architectural differences in blood vessels in the white matter adjacent to the ventricular angles could explain why demyelination is so common at these sites^114^. In addition, the different patterns of cortical demyelination can also be traced to the architecture of veins^115^. Microvessels in the meninges in our study contained PTEs, and inflammation precipitated by toxicants in these blood vessels could be responsible for the meningeal inflammation and subpial cortical demyelination often found in MS patients^116^. As well as being present in endothelial cells, PTEs were found in astrocytes and pericytes, all cells needed to maintain the blood–brain barrier^60,117^. PTEs such as mercury and silver have been shown to affect the blood–brain barrier^104,118^, which is damaged in MS^60^.

Recently, attention has again been directed to the perivascular space of post-capillary venules as an initiator of autoimmune inflammation in MS^18^. Incidentally, this space is probably the site referred to in the seminal paper of Dawson in 1915 as the “lymphatic channels surrounding the veins”^112^. Our finding of PTEs attached to macrophages in the perivascular space suggests that metals such as mercury that bind to sulfhydryl groups on macrophages and white blood cells could activate these cells and initiate the autoimmune inflammation seen in acute MS plaques^52,101,119–122^.

The two regions of the brain where microvessels most avidly took up PTEs were the anterior pons and the lateral geniculate body. These are the two regions that frequently undergo osmotic demyelination in central pontine and extrapontine myelinolysis^123^, which suggests an anatomical neurotoxic link exists between MS and osmotic demyelination.

The nature of the threads connecting brain microvessels that we detected on autometallography in people with widespread PTEs is unclear, since we could not find reference to them elsewhere. They could possibly be involved in structural stabilisation of the brain capillary network. These threads may be visible only on autometallography because they have a high sulfhydryl content, as does the adjacent basal lamina^124^, so that they bind toxic metals such as silver and mercury that have an affinity for sulfhydryl groups.

Different types of astrocytes, especially in white matter, in regions of the brain not affected by MS plaques, contained PTEs. It has been suggested, based on findings in a man who injected himself with metallic mercury, that mercury within the various types of grey and white matter astrocytes could be related to the patterns of demyelination seen in MS^33^. The magnitude of plaques could be determined by toxicants moving through chains of fibrous astrocytes, and through the long processes of interlaminar and polarised astrocytes^33^, which we found in the current study to be more widespread than initially described^125^. Once within astrocytes, PTEs could pass into oligodendrocytes via gap junctions^126^ and cause apoptosis and autoimmune inflammation^127^. Furthermore, astrocytes affected by PTEs could produce toxins which damage oligodendrocytes^128^.

In the white matter of our samples, small PTE clusters were seen in oligodendrocytes in people with widespread PTEs. These oligodendrocytes were usually found close to PTE-containing microvessels. Not all white matter tracts had PTE-containing oligodendrocytes, reinforcing the concept that the human brain contains different populations of oligodendrocytes^129^. Oligodendrocytes contain considerable amounts of iron^73^, and environmentally-derived PTEs could interact with iron to increase oxidative stress in oligodendrocytes^69^. PTEs in oligodendrocytes have also been seen in Parkinson’s disease^65^, suggesting that demyelination occurs only in people who have predisposing factors to damage from these toxicants.

Neurons in the thalamus and cerebellar dentate nucleus contained PTEs in people with widespread brain PTEs. Thalamic volume is reduced in early MS and is related to clinical disability^130^. While some of this atrophy may be due to white matter damage to thalamic tracts^131^, PTEs in thalamic neurons, which have also been seen in Parkinson’s disease^65^, could play a part. Similarly, PTEs in cerebellar dentate neurons could contribute to the cerebellar atrophy and ataxia commonly found in MS^132^.

The finding of bacterial toxins in the cerebrospinal fluid (CSF) of people with MS^133^ has re-focused attention on the possibility that toxins in the CSF could be responsible for attacks of demyelination, an idea that was first put forward more than a century ago^112^. In our study, lead and nickel were often seen in the CSF-containing leptomeninges covering the pons, or in the adjacent subpial tissue. These toxic metals in the CSF could penetrate the nearby parenchymal tissue, raising the possibility that toxicants in CSF could be a reason why large strips of subpial cerebral cortex undergo demyelination in MS^9^ (Fig. 1), and why subpial white matter plaques are common in the brain stem (Fig. 1) and spinal cord, since unlike the cerebrum, white matter is on the surface of the brain stem and spinal cord. Lead and nickel were sometimes seen in subependymal tissue surrounding the CSF-containing fourth ventricle in the pons. High levels of these metals in the ventricular CSF could pass through the ependyma and enter the surrounding tissue. This could explain why MS plaques are so common in periventricular regions of the brain^9^ (Fig. 1). However, these metals were occasionally seen at cut surfaces, and we cannot be certain that some artefactual edge effect may be present in these samples, so further toxic metal bio-imaging, including the cerebral lateral ventricles, is needed to confirm this finding.

Recent advances in the immunology of the perivascular space^18^, as well as factors such as the movement of toxicants into and out of the brain^50^, astrocyte-oligodendrocyte interactions^126,128^, oligodendrocyte apoptosis^12^, and neuroinflammation^15^, together with our findings on the distribution of toxicants in MS brains, suggest that PTEs in locus ceruleus neurons could cause a localised impairment in the blood–brain barrier, that would allow circulating PTEs to enter the perivascular space, activate white cells, and damage the blood–brain barrier. In prolonged PTE exposure, PTE entry into the perivascular space from two different directions (from both the blood and from astrocytic/ glymphatic clearance) would increase the toxicant load in the perivascular space. PTEs could then enter astrocytes and oligodendrocytes, resulting in oligodendrocyte apoptosis and demyelination, and myelin debris and PTEs would provoke an autoimmune inflammatory response.

The stage for PTE-induced demyelination could be set by increased genetic susceptibility to PTE toxicity or autoimmunity^134^, an increased tendency to metal-induced autoimmunity from low sun exposure^135^ and vitamin D levels^136^, and enhanced autoimmunity from Epstein-Barr virus infection^137,138^. After an initial exposure to PTEs, or after an accumulation of several PTEs, damage to locus ceruleus neurons could impair the blood–brain barrier and allows PTEs to enter white and grey matter perivascular spaces, from where circulating toxicants could be taken up by astrocytes and oligodendrocytes. Toxic interactions between PTEs, or between PTEs and essential elements such as iron, together with displacement of essential metals by PTEs^10,49,51^, could cause oligodendrocyte apoptosis, demyelination, and autoimmune inflammation. With repeated exposures to PTEs, the cycle could be repeated, given rise to relapsing–remitting MS. Progressive MS^139,140^ might arise from PTEs entering neurons or the edge of expanding plaques^20,141^, from recurrent PTE-induced cortical demyelination, or from organic PTEs being slowly demethylated in the brain to more toxic inorganic forms^100^.

This study has several limitations. (1) We were not able to assess the presence in the brain of all the many known PTEs^27^, so other PTEs may play a part in MS pathogenesis. Our findings, however, suggest metal toxicants other than the ones we identified could use the same pathways to enter oligodendrocytes. (2) No demographic or occupational data were available to estimate sources of PTEs, such as the presence of dental amalgams or the frequency of fish consumption^142^. (3) The frontal motor strip was not available to look for PTEs in corticomotor neurons. Corticomotor neurons are a frequent target of metal toxicants^33^, and toxicant-affected corticomotor neurons could be one factor behind the fatigue often experienced by MS patients^143^. (4) Data on cigarette smoking, a risk factor for MS^6^, were not available, so we were not able to assess if toxic metals contained in cigarette smoke^144^ could be contributing to the PTE burden within MS brains.

In conclusion, we found that more people with MS than controls had widespread metal toxicants in their brains, and that combinations of toxic metals were more common in MS than control brains. The cellular distribution of these toxicants, and their toxic properties, support the hypothesis that environmental toxicants play a role in MS. This work provides indirect evidence for a role for toxicants in MS, but future efforts to find genetic susceptibilities to these combinations of environmental toxicants, possibly using a Drosophila model^145^, could uncover further predispositions to MS. In the meantime, our findings suggest that a precautionary approach to reducing the risk of MS would be to limit as much as possible occupational and domestic exposure to toxic metals, take steps to reduce the emissions of toxic metals into the atmosphere from burning fossil fuels, reduce intake of food containing toxic metals (such as mercury in large fish like shark or swordfish), stop smoking cigarettes, re-assess the use of toxic metals in nanoparticles, and consider options other than toxic metal-containing materials for dental restorations.

## Supplementary Information

Supplementary Information 1.

Supplementary Information 2.

## Data Availability

All data generated or analysed during this study are included in this published article and its supplementary files.
