# Supplementary material for: Potentially toxic elements in the brains of people with multiple sclerosis
Source: Sci Rep. 2023 Jan 12;13:655. doi: 10.1038/s41598-022-27169-9 (PMC9837144; doi:10.1038/s41598-022-27169-9)
Supplement: Supplementary file 1 — Supplementary Information 1. [file 41598_2022_27169_MOESM1_ESM.docx]

Potentially toxic metals in the brains of people with multiple sclerosis (SREP-22-02660)

Supplementary information

**Supplementary Fig S1.** Autometallography of control brain CN12. **(a)** A branching microvessel in the anterior pons contains ^AMG^PTEs. Small ^AMG^PTE granules are present in cell bodies of oligodendrocytes (arrow). **(b)** Microvessels (open ­­arrowheads) in the lateral geniculate nucleus contain ^AMG^PTEs. Some microvessels are connected by thin AMG-positive threads (arrows). Neuronal cell bodies (closed arrowheads) contain brown lipofuscin but not ^AMG^PTEs. **(c)** ^AMG^PTEs are prominent in microvessels (arrowheads) and numerous glial cells (seen as small black paranuclear dots) within the white matter (WM, above dashed line) of the inferior olive in the medulla oblongata. No significant ^AMG^PTE is present in glial cells in the adjacent inferior olivary nucleus grey matter (GM, below dashed line), or in grey matter blood vessels or neuronal cell bodies (arrows). **(d)** In the white matter within the inferior olive of the medulla oblongata, ^AMG^PTE granules are present in the cytoplasm of numerous astrocytes (arrowheads) and oligodendrocytes (arrows), seen clearly in the enlarged inset. **(e)** In the putamen, numerous glial cells in white matter (WM, within dashed outline) contain ^AMG^PTE granules, seen as small black paranuclear dots. No glial cells or neuronal cell bodies (arrows) in the surrounding grey matter (GM) contain ^AMG^PTEs. ­**(f)** Most neurons in the cerebellar dentate nucleus grey matter (GM) contain cytoplasmic ^AMG^PTEs (open arrowheads, also in enlarged inset), whereas glial cells are ^AMG^PTE-free. In contrast, many glial cells in the white matter (WM, below dashed line) internal to the dentate nucleus contain ^AMG^PTEs (arrows). All AMG/H.

**Supplementary Fig S2**. LA-ICP-MSI of MS pons samples. AP anterior pons, PP posterior pons, MS multiple sclerosis ID, Dashed circles: position of locus ceruleus. The results are summarised in Table 1.

**Supplementary Fig S3**. LA-ICP-MSI of control pons samples. AP anterior pons, PP posterior pons, CN control ID. Dashed circles: position of locus ceruleus. The results are summarised in Table 1.
