# Supplementary figures and images for: Potentially toxic elements in the brains of people with multiple sclerosis
Source: Sci Rep. 2023 Jan 12;13:655. doi: 10.1038/s41598-022-27169-9 (PMC9837144; doi:10.1038/s41598-022-27169-9)

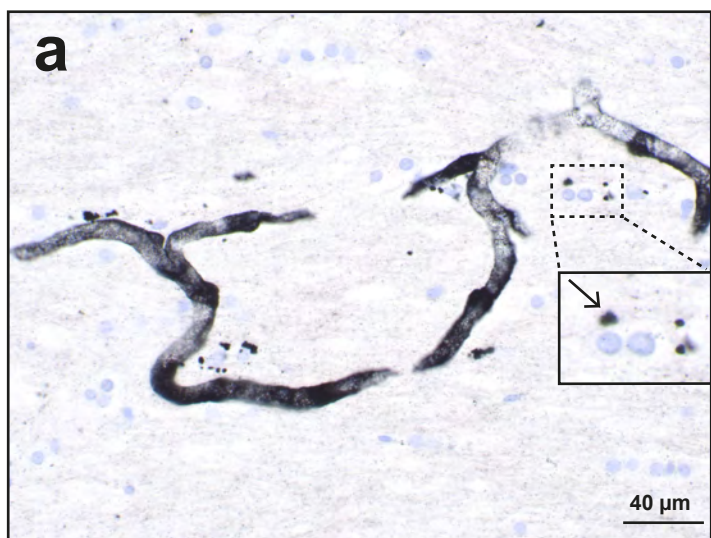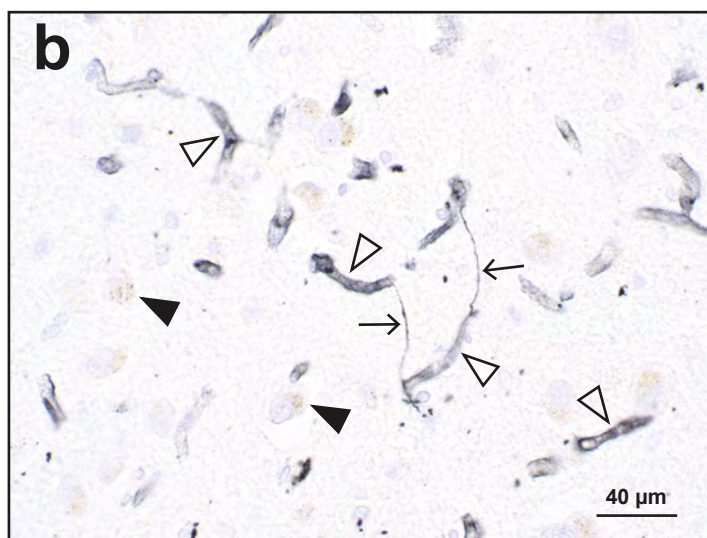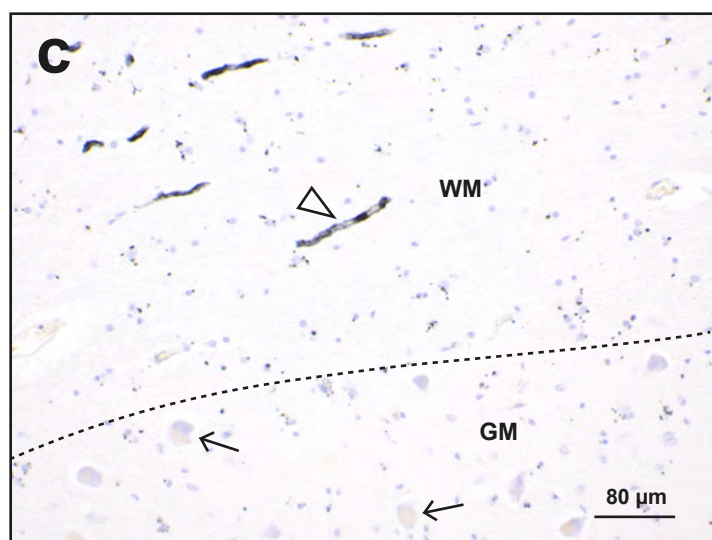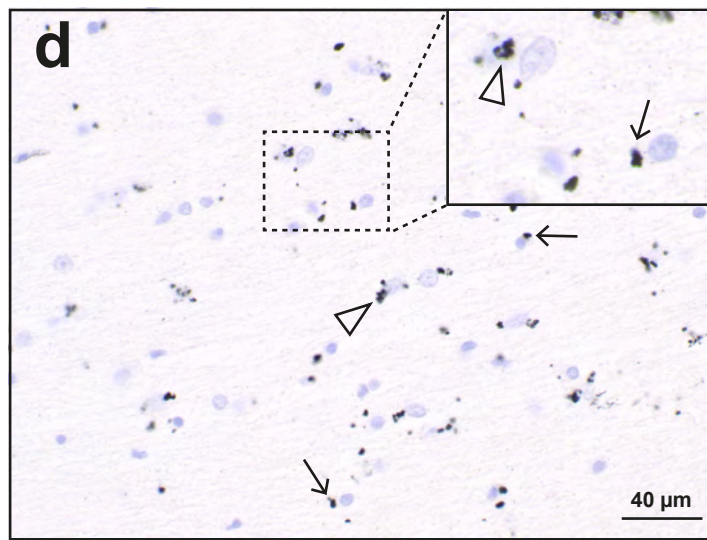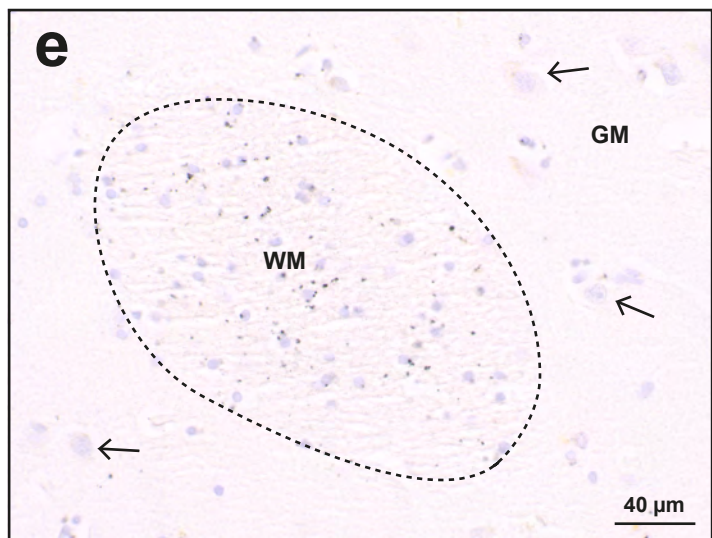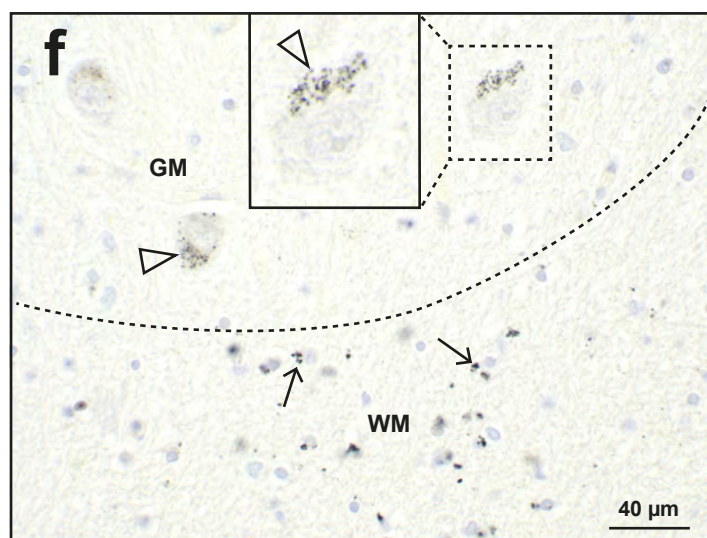

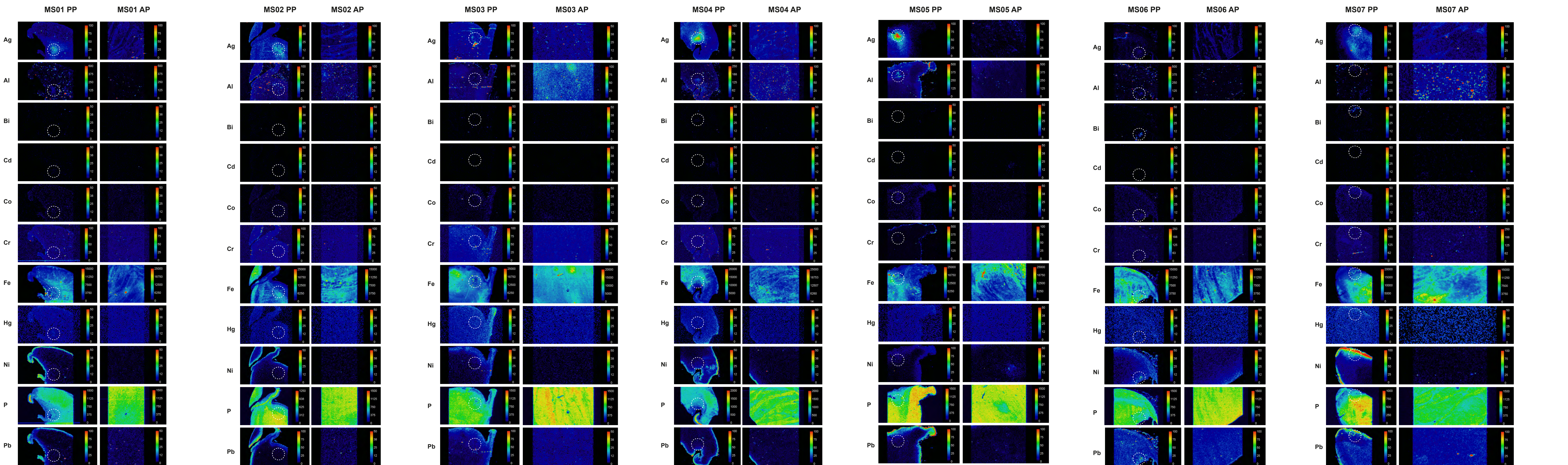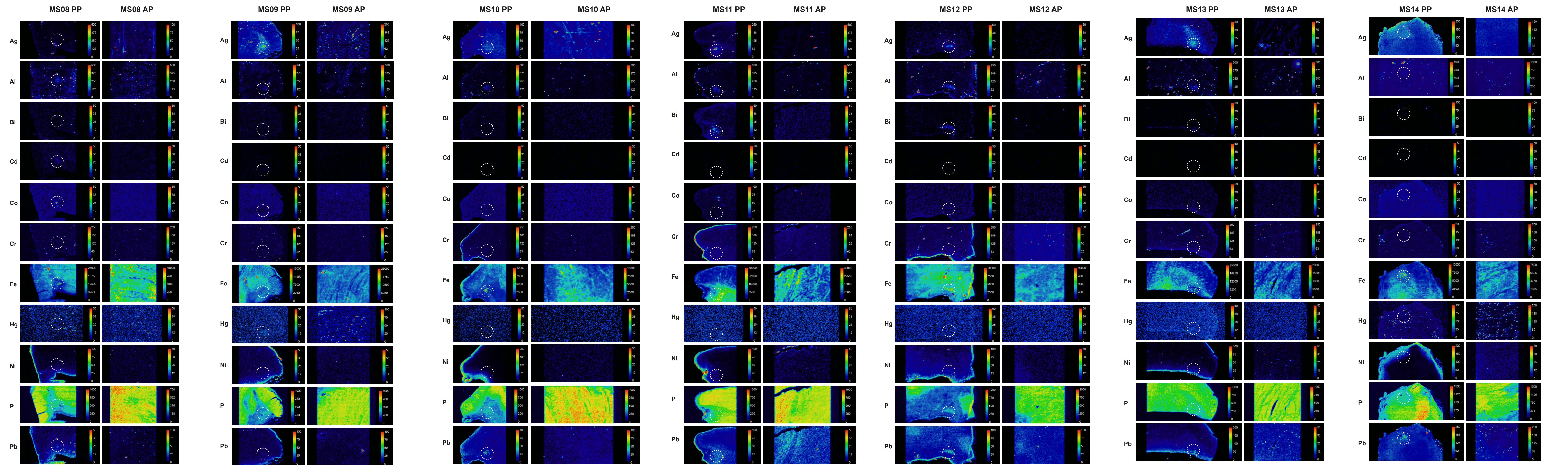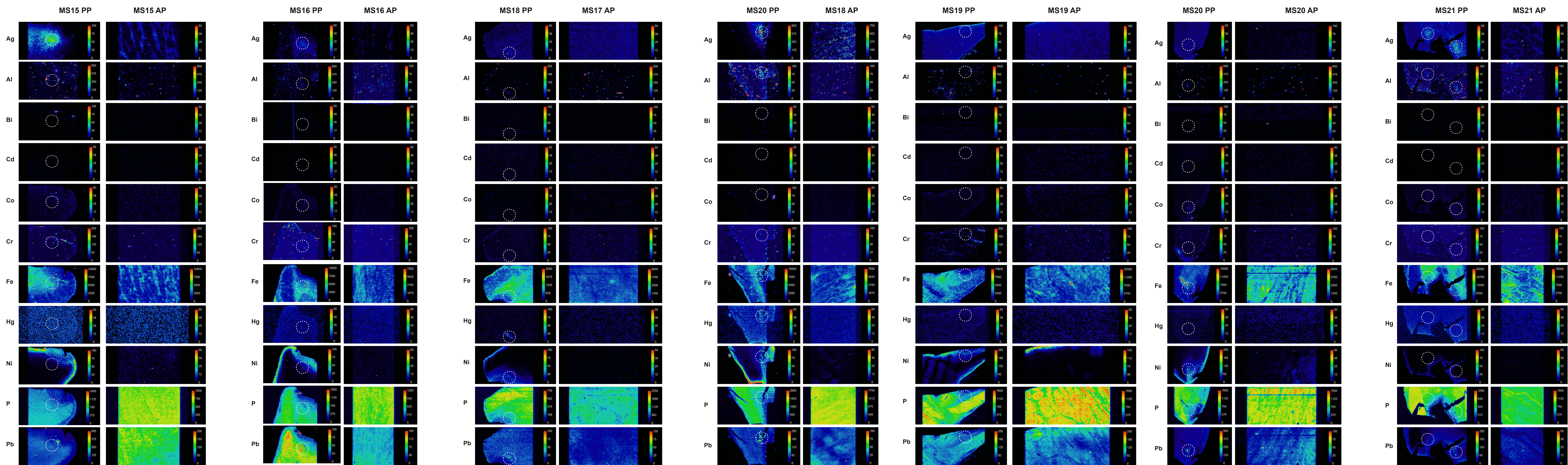

Supplement: Supplementary file 2 — Supplementary Information 2. [file 41598_2022_27169_MOESM2_ESM.pdf]
